# Clinical Outcomes of Patients with Achalasia Following Pneumatic Dilation Treatment: A Single Center Experience

**DOI:** 10.3390/jcm14155448

**Published:** 2025-08-02

**Authors:** Viktorija Sabljić, Dorotea Božić, Damir Aličić, Žarko Ardalić, Ivna Olić, Damir Bonacin, Ivan Žaja

**Affiliations:** 1 Institute of Emergency Medicine of Šibenik-Knin County, Narodnog Preporoda 1, 22000 Šibenik, Croatia; v.sabljic97@gmail.com; 2 Department of Gastroenterology and Hepatology, University Hospital Split, Spinčićeva 1, 21000 Split, Croatia; dalicic@gmail.com (D.A.); zardalic@gmail.com (Ž.A.); ivna1211@gmail.com (I.O.); damir.bonacin@yahoo.com (D.B.); ivan.zaja@yahoo.com (I.Ž.); 3 School of Medicine, University of Split, Šoltanska 2A, 21000 Split, Croatia; 4 Department of Health Studies, University of Split, Ruđera Boškovića 35, 21000 Split, Croatia

**Keywords:** pneumatic dilation, achalasia, dysphagia

## Abstract

**Background/Objectives:** Pneumatic dilation (PD) is a widely used treatment modality in the management of achalasia. It is particularly relevant in regions where many centers lack access to advanced therapeutic modalities. Therefore, we aimed to assess the effectiveness and safety of PD in our local region. **Methods**: This study retrospectively analyzed patients with achalasia that underwent PD from 1/2013 to 12/2019. The diagnosis of achalasia was established on the grounds of clinical symptoms, radiological and endoscopic findings, and esophageal manometry. Data on patient’s clinical characteristics, dilation technique and postprocedural follow-up were collected and statistically analyzed. Procedure effectiveness was defined as the postprocedural Eckardt score ≤ 3. **Results**: PD significantly reduced frequency of dysphagia, regurgitation, and retrosternal pain (*p* < 0.001). Body-weight increased significantly one month and one year after the procedure (*p* < 0.001). The procedural success rate was 100%. No severe complications were reported. **Conclusions**: PD is an effective and safe treatment modality in the management of achalasia. The study limitations include a single center design with the small number of participants, not all of whom underwent manometry, gender disproportion, absence of non-responders, and a short follow-up.

## 1. Introduction

Achalasia is a primary motor disease of the esophagus, manometrically characterized by the loss of peristalsis and a non-relaxing lower esophageal sphincter (LES) [1]. Patients with achalasia develop an immune-mediated loss of esophageal enteric neurons, which results in the failure of both esophagogastric junction (EGJ) relaxation and esophageal body peristalsis. This physiological dysfunction leads to symptoms of dysphagia, regurgitation, chest pain, and weight loss [1,2]. Achalasia is diagnosed using the barium-swallow esophagogram, esophagogastroduodenoscopy (EGDS) and esophageal manometry. The disease usually affects patients from the third to the sixth decade, and is most common in the fourth decade [3].

Three achalasia subtypes have been described according to the Chicago classification, depending on the high-resolution manometry findings in the esophageal body: type I or classic achalasia with the low intraesophageal pressure, type II with pan-esophageal pressurization, and type III with high-amplitude spastic contractions [4].

All available therapeutic methods focus on reducing the pressure gradient across the LES, since no treatment modality can reintegrate the muscular activity [5]. Relaxation of LES can be achieved by pharmacological agents, injected endoscopically (botulinum toxin) or taken orally (calcium channel blockers and nitrates), but their clinical effects are short-term [6]. The leading procedures for achalasia treatment include pneumatic dilation (PD), peroral endoscopic myotomy (POEM), and surgical myotomy [7,8]. PD is an endoscopic method that forces the hypertonic LES by intraluminal dilation of a pressurized noncompliant balloon [9]. Potential complications of PD include bleeding, chest pain, and perforation, which occurs in 2.9% to 4.3% of patients [10,11]. POEM is a newer, minimally invasive therapeutic method developed for the permanent treatment of achalasia [12]. It is the method of choice for treating patients with vigorous achalasia as it allows for a longer myotomy compared to laparoscopic Heller’s myotomy [13].

The majority of authors consider PD the first choice of treatment due to its high degree of effectiveness and low degree of morbidity and mortality, with the additional advantage of short hospitalization and low instrumental costs. Surgery is therefore usually reserved for patients who do not respond to PD [6,7]. European Society of Gastrointestinal Endoscopy (ESGE) recommends the use of graded PD protocol in achalasia, starting with a 30 mm dilation, followed by a 35 mm dilation 2–4 weeks after the initial procedure, and a subsequent 40 mm dilation in cases of insufficient LES relief [14].

The aim of this research was to investigate the effectiveness and safety of PD in the treatment of achalasia. We aimed to examine the association of patient characteristics with the achalasia symptoms before the procedure and to assess whether the outcomes are affected by the type of dilator used.

## 2. Materials and Methods

### 2.1. Patients

The study included patients diagnosed with achalasia who underwent pneumatic dilation between January 2013 and December 2019. All patients presented with clinical symptoms of dysphagia, regurgitation, retrosternal pain and weight loss, with the duration of symptoms varying among individuals. Eligibility criteria included diagnosis of achalasia, age > 18 years and no prior endoscopic or surgical interventions. Patients with esophageal tumors, Chagas disease and intestinal pseudo-obstruction mimicking achalasia were excluded from the study. All patients were informed about the procedure and signed informed consent.

### 2.2. Ethical Aspects

The study was conducted in accordance with the provisions of the Declaration of Helsinki and was approved by the Ethics Committee of the University Hospital of Split (500-03/22-01/196, 28 November 2022).

### 2.3. Study Design

This retrospective study was conducted at the Department of gastroenterology, University Hospital of Split. We included patients that underwent PD from January 2013 until December 2019 and were followed up for a year. The diagnosis of achalasia was established on the grounds of clinical symptoms, timed barium esophagogram (radiographs taken at 1, 2, and 5 min after swallowing 200 mL of dilute contrast), esophagogastroduodenoscopy (EGDS), and the absence of peristalsis with the impaired relaxation of LES (lowest pressure of 10 mm Hg during swallow-induced relaxation) at esophageal manometry.

Three subtypes of achalasia were defined depending on the manometry findings and according to the Chicago classification. Demographic and disease-specific information including age, gender, frequency, duration, and type of symptoms (dysphagia, regurgitation, retrosternal pain and weight loss) were collected. Disease severity was defined using the Eckardt symptom score (Table 1) [15]. Patients were additionally categorized depending on pain characteristics: location, intensity, direction of pain radiation, duration, relieving factors, and exacerbating factors.

### 2.4. Procedure

Patients fasted for at least eight hours before the procedure. Adequate sedation was achieved using midazolam (Midazolam, Kalcex, Riga, Latvia), fentanyl (Fentanyl Kalcex, Riga, Latvia) and propofol (Propofol, Fresenius Kabi, Bad Homburg, Germany), with continuous monitoring of vital signs. Two types of dilators were used: Rigiflex balloon dilator (Boston Scientific, Marlborough, MA, USA) up to 35 mm in diameter and Witzel balloon dilator (Wimed GmbH Medizintechnik, Berlin, Germany) up to 40 mm in diameter. The type of dilator used was based at the discretion of the endoscopist. The balloon was inflated with an initial pressure ranging from 150 mmHg to 190 mmHg. The pressure was gradually increased to 300 mmHg within one minute, up to three times in each individual procedure. Patients were allowed to drink fluids two to three hours after the procedure and started consuming semi-liquid foods after 24 h. They were discharged on the same day or the day after the procedure, depending on their clinical condition.

### 2.5. Follow-Up

Patient follow-up lasted for a year, with the first clinical and endoscopic check-up one week after the procedure. Additional follow-ups were performed one month and one year after the procedure. The Eckardt symptom score was used to assess the presence of achalasia symptoms. A total Eckardt score ≤ 3 was used to define treatment success, in line with the recent achalasia trials [15,16]. All collected data were organized in Excel sheets and statistically analyzed.

### 2.6. Statistical Analysis

Statistical analysis was performed using Jamovi software (Jamovi Project, Sydney, Australia), version 2.3. Categorical variables were presented as count and frequency. Continuous quantitative variables were presented as mean (M) ± standard deviation (SD), minimum (min), and maximum (max). Pearson correlation coefficients were calculated among the preprocedural patient characteristics. Since the data distribution for continuous variables was normal, *t*-test and repeated measures analysis of variance were used to assess differences among study groups. An independent sample *t*-test was conducted to evaluate the difference in effectiveness of PD based on the type of dilator used and repeated measure analysis was used to examine differences in achalasia symptoms before and after the procedure. Statistical significance level was set at *p* < 0.05.

## 3. Results

### 3.1. Patient Characteristics

A total of 23 patients (17 females, mean age 61.48 ± 11.40 years) were included in the study. The average symptom duration was 22.48 ± 20.53 months and all of the included patients presented with symptoms of dysphagia, regurgitation, retrosternal pain, and weight loss. Data on patient demographic characteristics, as well as the achalasia symptoms are presented in Table 2.

Various types of pain were observed among the subjects: a sensation of heaviness (17 patients), stabbing pain (2), discomfort (2), and a burning sensation (2). Regarding the duration of pain, nine patients experienced pain that lasted longer than 10 min, nine patients reported short episodes of pain (lasting less than 5 min) and the remaining five patients had episodes of pain lasting for 5–10 min.

Depending on the number of painful episodes per week, patients were categorized into three groups: <5 episodes, 5–10 episodes, and >10 episodes per week. According to our data, six participants belonged to group 1, ten participants to group 2, and seven patients to group 3.

The results also indicated that diet and fluid intake reduced the sensation of pain, while the intake of solid food resulted in exacerbation of painful episodes. Regarding the endoscopic changes, all subjects had nearly identical changes at the time of the initial upper endoscopy, including esophageal dilation, spastic contractions, and an abundant quantity of liquid content in esophagus. A total of thirteen participants were classified according to the Chicago classification. Of these, two participants (15.4%) were classified as type I, six participants (46.2%) as type II, and five participants (38.4%) as type III.

### 3.2. Correlation of Preprocedural Patient Characteristics

In order to examine the correlation between patient’s general characteristics and symptoms of achalasia, Pearson correlation coefficients were calculated.

The female gender was found to be associated with the more frequent preprocedural reports of dysphagia (P = 0.455, *p* = 0.029), while the higher age was linked to a less frequent occurrence of regurgitation symptoms (P = 0.473, *p* = 0.023). Higher frequency of regurgitation was associated with the longer duration of symptoms (P = 0.444, *p* = 0.034), while the longer duration of pain per episode was associated with the lower number of painful episodes per week (P = −0.589, *p* = 0.003). Table 3 provides correlation matrix between the observed variables.

### 3.3. Postprocedural Outcomes

Symptoms of retrosternal pain and dysphagia subsided completely 4 weeks after the treatment in all patients (100%). Significantly lower frequency of regurgitation was also reported (Table 4).

**Scheme 1:**
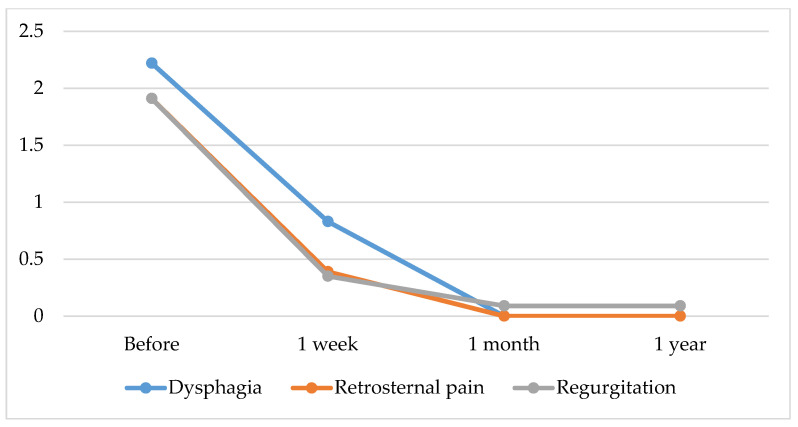
Changes in Eckardt scores following the PD (N = 23).

Changes in Eckardt scores in the follow-up period are graphically presented in Scheme 1.

The results of the repeated measures analysis of variance for frequency of dysphagia (*F = 48.9, df = 3/22, p < 0.001*), regurgitation (*F = 69.8, df = 3/22, p < 0.001*) and retrosternal pain (*F = 62.6, df = 3/22, p < 0.001*) presented as Eckardt scores, imply statistically significant changes in symptom frequency in the period before and after the procedure (Table 5).

The results of the repeated measures analysis of variance for body weight (*F = 30.2, df = 3/22, p < 0.001*) suggest that there has been a significant change in the body weight before and after the procedure. We found a statistically significant increase in body weight one month compared to one week after the procedure, and one year compared to both one week and one month after the procedure (Table 6).

To examine the difference in the final Eckardt score, a repeated measures analysis of variance was conducted *(F = 229, df = 3/22, p < 0.001)*, indicating a significant change over the follow-up period. One week after the procedure, as well as one month, and one year after the procedure, we found a reduction in the final Eckardt score compared to the preprocedural value. Furthermore, we found an additional decrease one month and one year after the procedure when compared to values detected one week after the procedure. However, we found no significant difference in Eckardt score between periods of one month and one year after the procedure. All patients (100%) had successful PD treatment that was defined as the final Eckardt score ≤ 3.

**Scheme 2:**
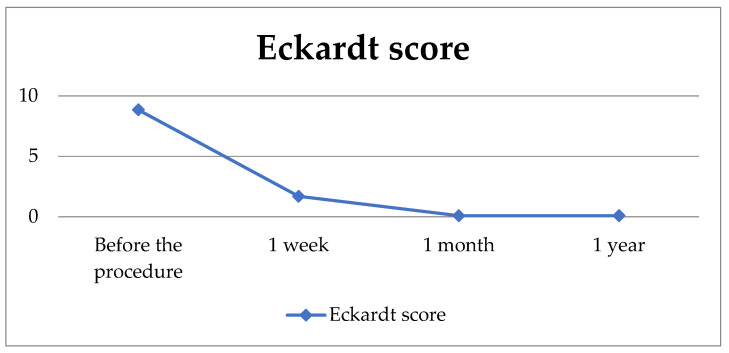
Graphical illustration of Eckardt score values during the follow-up period.

Graphical illustration of the final Eckardt score values during the one-year follow-up is presented in Scheme 2.

Based on the recorded complications, 19 participants (82.6%) reported pain that subsided spontaneously without the use of analgesics. In four participants, the pain decreased after the use of analgesics. There were no cases of bleeding or esophageal perforation in our cohort.

### 3.4. Procedure Efficiency Regarding the Chicago Classification

**Scheme 3:**
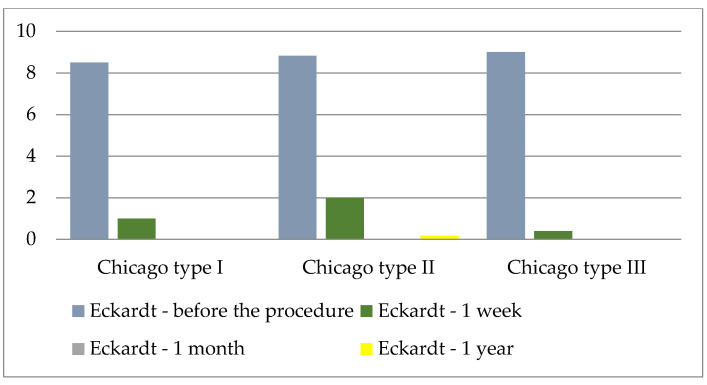
Graphical illustration of the average Eckardt score values in relation to the Chicago classification (N = 13).

Among the 23 patients included in the study, 13 were classified according to the Chicago classification due to limited access to high-resolution manometry. The absence of this system prevented the classification of the remaining patients. The graphical illustration of the average values of the Eckardt score in relation to the Chicago classification is presented in Scheme 3.

In order to determine whether there is a difference in the Eckardt scale with regard to the Chicago classification, ANOVA for repeated measurements was calculated, and no statistically significant difference was found (Table 7).

### 3.5. Procedure Efficiency Regarding the Type of Dilator

Regarding the procedure itself, various dilators were used in this study. The most frequently used was the Rigiflex 30 mm (11 patients), while Rigiflex 35 mm was used in 8 patients, and the Witzel 40 in 4 patients. In two cases, the procedure was repeated after one week. One participant was referred for Heller’s myotomy, while a 40 mm Witzel dilator was used for the other participant.

To examine the effectiveness of the PD procedure depending on the type of dilator used, an independent sample *t*-test was conducted. Since the groups differed in the number of participants, a Levene’s test for homogenicity of variances was conducted. There was no significant difference in the reduction in achalasia symptoms based on the type of dilator used.

## 4. Discussion

In our cohort of patients treated for achalasia, we found an outstanding performance of PD. All patients had successful treatment response in terms of symptomatic improvement presented in Eckardt scale, without development of any severe complications. Although these findings are consistent with previously published data, they are particularly relevant in our local context, where many centers lack access to advanced therapeutic modalities.

According to our study, female patients with achalasia suffer more frequently than male patients from symptoms of dysphagia, while older patients have lower occurrence of regurgitation. In accordance with our results, a study by Xu et al. [17] confirmed that male patients with achalasia more frequently complain of heartburn and regurgitation, whereas female patients complain of more severe dysphagia. In their systematic review, Katsumata R. et al. [18] reported similar sex distribution among the three achalasia subtypes, although one study noted higher prevalence of men in type III [19]. In our cohort that consisted of 23 patients, 13 of them were classified according to the Chicago classification with the following gender distribution: Chicago I: 1 female (F) and one man (M), Chicago II: 4F and 2M, Chicago III: 4F and 1M.

Katusmata R. et al. [18] reported no gender differences regarding the presence of symptoms or treatment response in each subtype. Similarly, Aljebreen et al. [20] found no statistically significant impact of age or gender in prediction of symptom improvement according to the Eckardt score. Karamanolis G. et al. [21] reported that female patients had better outcomes following the single dilation. We found no differences in treatment response depending on age or gender since all of the treated patients had a positive treatment response defined as the final Eckardt score ≤ 3.

We explored the response to PD in three subtypes of achalasia and found no significant variations. Following the PD, we found a statistically significant reduction in the frequency of the most commonly reported symptoms (dysphagia, regurgitation, and retrosternal pain) in patients with achalasia, as well as the significant reduction in Eckardt score one week after the procedure. Furthermore, a further reduction in dysphagia was observed one month and one year after the procedure. However, there were no further reductions in the frequency of regurgitation or retrosternal pain one month or one year after the procedure. Our data indicate that it is reasonable to expect at least one year of symptom resolution following the PD treatment.

We demonstrated an increase in body weight one month and one year after the procedure. Interestingly, Patel et al. [22] reported opposite results. The authors found that 43% of patients who initially reported weight loss denied regaining their weight after undergoing achalasia treatment [22]. Objective markers such as comparing pre- to post interventional body weight have not been used in other clinical studies to evaluate treatment efficacy. It is also unknown why certain patients with achalasia are prone to a more significant weight loss on presentation when compared to others.

Our results indicate the effectiveness of PD as a treatment method for achalasia in reducing symptoms of dysphagia, regurgitation, and retrosternal pain. This technique has the advantages of wide availability and affordability and does not require hospitalization when compared to other methods. However, proving the superiority of one achalasia treatment method over another presents a challenge. The prevalence of the disease is low, and randomized controlled clinical trials are rare, especially in terms of long-term outcomes [23]. Therefore, one of the main questions in evaluating PD as a treatment for achalasia is the durability of response. In general, significant symptom improvement has been observed five years after PD in more than 50% of patients. Certain studies demonstrated that more than a third of patients continue to benefit from a single PD up to 10 years after the treatment. These results are in line with reports documenting patients with long-term and well-controlled achalasia symptoms 15 to 20 years after the procedure [24,25,26].

The effectiveness of PD may be affected by various factors, including the type of balloon (pneumatic or hydrostatic; so-called high-compliance or low-compliance dilators), balloon diameter (30, 35 or 40 mm), the need for fluoroscopic guidance, the degree of balloon inflation (partial or complete), the duration of balloon inflation, and criteria for discontinuing balloon distension, such as the occurrence of pain. Although there are different opinions on the optimal balloon diameter and the number of sessions required to achieve desired results, this can also pose a challenge in the analysis of research results [23].

We used three types of dilators: Rigiflex 30 mm in 11 patients, Rigiflex 35 mm in 8 patients, and the Witzel 40 in 4 patients. Our protocol was based on the balloon inflation with an initial pressure ranging from 150 mmHg to 190 mmHg, which was then gradually increased to 300 mmHg within one minute, up to three times in each individual procedure. Although the two times balloon inflation is considered the standard, several authors described the three times inflation as a successful variant [27,28,29]. According to ESGE, there are no specific recommendations regarding the number of balloon inflations, pressure targets nor duration of inflation per procedure. However, ESGE recommends a graded PD protocol, which was not consistently implemented mostly due to symptomatic relief after the initial dilation [14].

Preliminary observations from our study did not show a statistically significant difference in effectiveness between the two types of dilators (Rigiflex vs. Witzel), which is in accordance with other studies [24,30]. Unlike the majority of studies [31] that studied the Rigiflex dilator, patients in the study conducted by Tuset et al. [32] were treated with the Witzel dilator (40 mm in diameter), and a similar rate of complications was registered. These results require further analysis to confirm any long-term differences between the two types of dilators. Hoeij’s research [30] presented PD as the safest and most effective dilation method, regardless of the type of dilator used, with the recommendation of performing the dilation by gradually increasing the balloon size. According to the most important relevant works in this research area, a gradual approach is the most important guidance when performing dilation [30]. Although the conventional clinical approach involves a “graded dilation” strategy that allows progression to larger balloons if needed, certain trials defined treatment success as the response to a single dilation, and progression to a larger balloon was considered as a treatment failure. The response to graded PD is the most relevant outcome for clinical practice. Certain studies did not clarify whether the reported clinical success was accomplished after a single or with graded dilations; therefore, further studies on larger sample size and more precise approach should be conducted [33].

Our study reported a 100% response rate according to the Eckardt score (≤3), which is similar to other studies with reported success rates over 90%. Tenlik I. et al. [34] conducted a study on 143 geriatric and 150 non-geriatric patients who were treated with PD, and found success rates of 99% and 94%, respectively. Similarly, 91% of patients remained symptom-free during the follow-up period (median 48 weeks) in the study on 72 patients treated for achalasia with graded dilations [35]. However, other authors reported lower success rates ranging from 54 to 69% [36,37]. The randomized multicenter clinical trial that included 133 patients with the newly diagnosed achalasia proved a significantly higher efficiency of the peroral endoscopic myotomy (POEM) (92%) compared to PD (54%), *p* < 0.001 [37].

In Torresan’s studies, whose results were in line with the excellent success rates in preventing symptom recurrence, a significant occurrence of reflux as a complication of the procedure was noted [38]. On the contrary, in our study, the absence of complications in the short term confirmed and extended previous evidence of the safety of PD. Esophageal perforation is the most serious complication of PD [39]. Recent studies reported the rate of esophageal perforation conducted with a Rigiflex balloon dilator of 0.5–3.0% [40,41]. However, no patients in our study suffered from esophageal perforation during treatment with PD. Our results confirm that our treatment principle characterized by the low number of repeated dilations and low inflation pressure balloon is therapeutically safe and effective.

Regarding other therapeutic options, a randomized controlled trial with the 5-year follow-up published by Kuipers et al. [42] showed that POEM is associated with the significantly higher efficacy when compared to PD as initial treatment of therapy-naive patients with achalasia. Also, the risk of re-admission and resource utilization in the USA is higher in patients with achalasia undergoing PD in comparison to POEM [43]. Therefore, POEM may be proposed as an initial treatment option for patients with achalasia. However, POEM is a challenging, long learning curve method, usually implemented only in specialized centers. ESGE recommends consideration of gastric POEM in carefully selected patients because it is an emerging procedure with limited data on effectiveness, safety, and durability. It should therefore be performed in expert centers [14].

The study has several limitations, including a small number of participants, not all of whom underwent manometry, a gender disproportion with a predominance of female participants, an absence of non-responders, a single center design of the study and a relatively short follow-up period of one year. Additionally, the retrospective design of the study and potential selection bias due to the non-random assignment of patients could have potentially overestimated the treatment efficacy. Prospective studies are needed to confirm these results and reduce potential biases.

## 5. Conclusions

To conclude, our results confirm the effectiveness and safety of PD in alleviation of achalasia symptoms and support the use of PD as a relevant therapeutic option. PD is a non-expensive, procedurally relatively simple method, with good efficacy and low complication rates, widely implemented worldwide. Ideally, all of the treatment options should be discussed with treatment-naive patients with achalasia and a shared decision should be made.

Although the results of our study are consistent with previously published data, they hold particular relevance in our local context. Many centers, especially in resource-limited regions, lack access to advanced therapeutic options such as POEM, making PD a valuable and accessible alternative for achalasia treatment.

## Figures and Tables

**Table 1:** Achalasia severity according to the Eckardt score [15].

Symptom	Severity	Score
Weight loss (kg)		
	none	0
	<5 kg	1
	5–10 kg	2
	>10 kg	3
Dysphagia		
	none	0
	occasional	1
	every day	2
	during each meal	3
Retrosternal pain		
	none	0
	occasional	1
	every day	2
	during each meal	3
Regurgitation		
	none	0
	occasional	1
	every day	2
	during each meal	3

**Table 2:** The patient characteristics and symptoms (N = 23).

Variable		N (%)	*M*	SD	Min.	Max.
Gender	M	6 (26.1)				
	F	17 (73.9)				
Dysphagia		23 (100)				
Regurgitation		23 (100)				
Retrosternal pain		23 (100)				
Weight loss		23 (100)				
Age (years)			61.48	11.40	39	83
Duration of symptoms (months)			22.48	20.53	2	75

N: sample size; M: male; F: female; *M*: mean; SD: standard deviation; Min.: minimum; Max.: maximum.

**Table 3:** Correlation matrix between general patient characteristics and achalasia symptoms.

Variable	Gender	Age (y)	Symptom Duration *	Dysphagia	Retrosternal Pain	Pain Duration **	Painful Episodes ***	Regurg.
Gender	1							
Age (y)	0.168	1						
Symptom Duration *	0.059	−0.199	1					
Dysphagia	0.455	0.025	−0.007	1				
Retrostenal Pain	−0.059	0.394	0.010	−0.039	1			
Pain Duration **	0.000	0.110	−0.115	0.137	0.167	1		
Painful Episodes ***	0.034	−0.023	0.351	−0.098	0.006	−0.589	1	
Regurg.	−0.194	−0.473	0.444	0.190	−0.011	−0.127	0.081	1

y: year, * months, ** minutes, *** per week, regurg: regurgitation.

**Table 4:** Achalasia symptoms according to their Eckardt scores before and after the pneumatic dilation (N = 23).

		Eckardt Score					
Symptom	Initial	1 Week	4 Weeks	1 Year	*F*	*df*	*p* *
Dysphagia	2.22 ± 0.74	0.83 ± 1.23	0.00 ± 0.00	0.00 ± 0.00	48.9	3/22	<0.001
Retrosternal pain	1.91 ± 0.90	0.39 ± 0.66	0.00 ± 0.00	0.00 ± 0.00	62.6	3/22	<0.001
Regurgitation	1.91 ± 0.79	0.35 ± 0.49	0.09 ± 0.29	0.09 ± 0.29	69.8	3/22	<0.001

Variables are presented as mean ± standard deviation. *F*: F-ratio; *df*: degrees of freedom; *p*: *p*-value. * ANOVA.

**Table 5:** Difference in dysphagia, regurgitation, and retrosternal pain frequency (presented as Eckardt scores) between different measurement points (N = 23).

Symptom	Time-Frame	*t*	*df*	*p*
**Dysphagia**				
Before the procedure	1 week	4.64	22	<0.001
	1 month	14.45	22	<0.001
	1 year	14.45	22	<0.001
1 week	1 month	3.22	22	0.024
	1 year	3.22	22	0.024
1 month	1 year	0.00	22	1.000
**Regurgitation**				
Before the procedure	1 week	7.24	22	<0.001
	1 month	10.50	22	<0.001
	1 year	10.50	22	<0.001
1 week	1 month	2.79	22	0.065
	1 year	2.31	22	0.183
1 month	1 year	0.00	22	1.000
**Retrosternal pain**				
Before the procedure	1 week	6.75	22	<0.001
	1 month	10.19	22	<0.001
	1 year	10.19	22	<0.001
1 week	1 month	2.86	22	0.055
	1 year	2.86	22	0.055
1 month	1 year	0.00	22	1.000

*t*: *t*-test with Bonferroni correction; *df*: degrees of freedom; *p*: *p*-value.

**Table 6:** The difference in the body weight between different measurement points (N = 23).

Body Weight		*t*	*df*	*p*
1 week	1 month	−6.54	2	<0.001
	1 year	−6.63	2	<0.001
1 month	1 year	−2.98	2	0.021

*t*: *t*-test with Bonferroni correction; *df*: degrees of freedom; *p*: *p*-value.

**Table 7:** ANOVA for repeated measurements for the Eckardt scale with regard to the Chicago classification.

	*F*	*df*	*p* *
Time measurement point	106.97	3/10	<0.001
Chicago class.	1.06	2/10	0.382
Time measurement point × Chicago class.	0.535	6/30	0.777

*F*: F-ratio; class: classification; *df*: degrees of freedom; *p*: *p*-value. * ANOVA.

## Data Availability

Data are available upon request.

## Abbreviations

The following abbreviations are used in this manuscript:

PD	Pneumatic dilation
*p*	*p*-value
LES	Lower esophageal sphincter
EGJ	Esophagogastric junction
EGDS	Esophagogastroduodenoscopy
POEM	Peroral endoscopic myotomy
ESGE	European Society of Gastrointestinal Endoscopy
*M*	Mean
SD	Standard deviation
Min.	Minimum
Max.	Maximum
N	Sample size
M	Male
F	Female
y	Year
Regurg.	Regurgitation
*F*	F-ratio
*df*	Degrees of freedom
ANOVA	Analysis of Variance
*t*	*t*-test with Bonferroni correction
class.	Classification
